# Supplementary material for: Mass Spectrometry-Based Top-Down Proteomics in Nanomedicine: Proteoform-Specific Measurement of Protein Corona
Source: ACS Nano. 2024 Sep 14;18(38):26024–36. doi: 10.1021/acsnano.4c04675 (PMC11440641; doi:10.1021/acsnano.4c04675)
Supplement: Supplementary file 2 — nn4c04675_si_002.pdf [file nn4c04675_si_002.pdf]

Supporting Information I

Mass spectrometry-based top-down proteomics in nanomedicine: proteoform-specific measurement of protein corona

Seyed Amirhossein Sadeghi,[1] Ali Akbar Ashkarran,[2] Qianyi Wang,[1] Guijie Zhu,[1] Morteza Mahmoudi,[2]* Liangliang Sun[1]*

[1]Department of Chemistry, Michigan State University, 578 S Shaw Lane, East Lansing, Michigan 48824, USA

[2]Department of Radiology and Precision Health Program, Michigan State University, East Lansing, Michigan 48824, USA

* Corresponding Authors.

Morteza Mahmoudi: mahmou22@msu.edu

Liangliang Sun: lsun@chemistry.msu.edu

Experimental section

TDP analysis of a standard protein mixture for technique validation

To evaluate whether our protein corona sample preparation procedure leads to significant changes in proteoforms (i.e., artificial modifications) or not, we employed a standard protein mixture as a model system containing small (ubiquitin, Ub), medium (myoglobin, Mb), and large (carbonic anhydrase, CA) proteins. The standard protein mixture was then analyzed by CZE-MS in two different conditions. In the first condition, the standard protein mixture was dissolved in 1% acetic acid (AA) and analyzed by CZE-MS directly. In the second condition, the standard protein mixture was processed using the same procedure as the protein corona sample, including dissolving in 0.4% SDS, incubating at 60 °C for 1.5 hours, cleaning up using buffer exchange, and analyzing by CZE-MS. The Agilent Q-TOF mass spectrometer (6545 XT) was used for this experiment and the basic CZE conditions are the same as the protein corona samples. The mass spectra of those proteins are shown in **Figure S5.**

Comparison of BUP and TDP regarding sensitivity

For TDP, intact bovine serum albumin (BSA) was dissolved in 100 mM ammonium bicarbonate (ABC) buffer to prepare a 2 mg/mL stock solution. The BSA sample was further diluted to 0.5, 0.75, and 1 mg/mL using the 100 mM ABC buffer. Each BSA solution was analyzed using CZE-MS in triplicate. The BSA intensity was plotted as a function of protein concentration to create the calibration curve of TDP, **Figure S6A**.

For BUP, a 1 mg/mL BSA solution in 100 mM ABC buffer (pH 8.0) was prepared. Then, 2 µL of 1 M dithiothreitol (DTT) was added to the protein solution for reduction at 95 °C for 10 minutes. After cooling, the protein solution was alkylated with 5 µL of 1 M iodoacetamide (IAA) for 30 minutes in the dark at room temperature. Trypsin (5 µg, Bovine pancreas TPCK-treated) was then added for protein digestion, which was carried out at 37 °C for overnight. The digestion process was stopped by adding formic acid to a final concentration of 0.6% (v/v). The sample was desalted using a Sep-Pak C18 Cartridge (Waters, Milford, MA) following the manufacturer's protocol. The eluate was lyophilized using a vacuum concentrator and re-dissolved in 100 mM ABC buffer (pH 8.0) to create a 1 mg/mL stock solution. The stock solution was diluted to 0.025, 0.065, 0.125, 0.25, and 0.5 mg/mL using the 100 mM ABC buffer, followed by CZE-MS analysis in technical triplicate. The intensity of two high-intensity peptides was plotted as a function of BSA digest concentration to produce the calibration curves of BUP, **Figures S6B** and **S6C**.

(A)

NPs	Size (nm)	SD (nm)	PDI	Zeta potential (mV)	SD (mV)
Bare NPs	78.9	0	0.028	-31.6	0.3
After protein binding	105.3	3.8	0.208	-14.7	2.9
After protein elution	92.9	0	0.042	-19.7	1.2

(B)

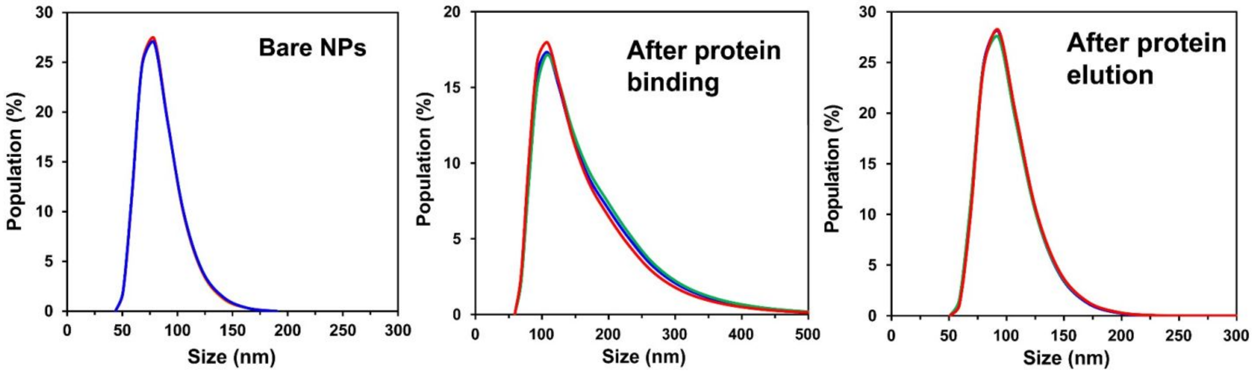

Figure S1. Characterization of different NPs. (A) Average size, polydispersity index (PDI), zeta potential, and the corresponding standard deviation values of protein corona coated NPs (all measurements are repeated three times, and the corresponding averages are reported). (B) Size distribution and the corresponding replicates of bare and protein corona coated NPs after protein binding and elution, respectively. The results are representative of 3 independent analyses.

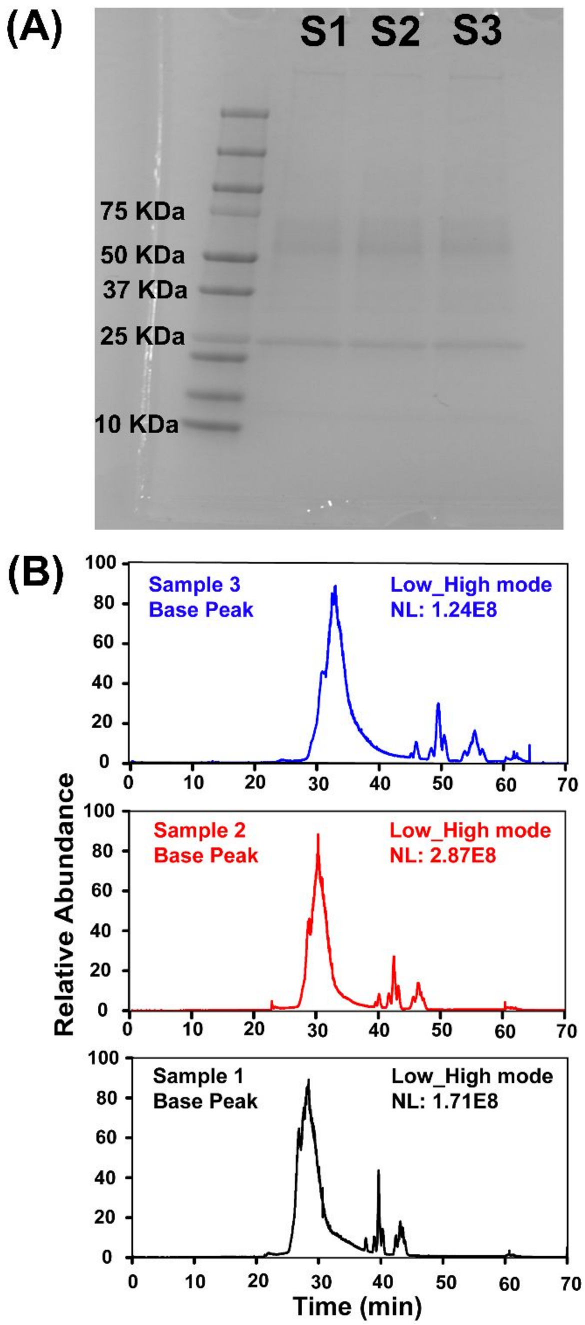

Figure S2. Reproducibility of CZE-MS/MS for measurements of large proteoforms. (A) SDS PAGE analysis of three protein corona samples (S1, S2, and S3) prepared in parallel. (B) Base peak electropherograms of the three protein corona samples after CZE-MS/MS analysis in “low-high” mode.

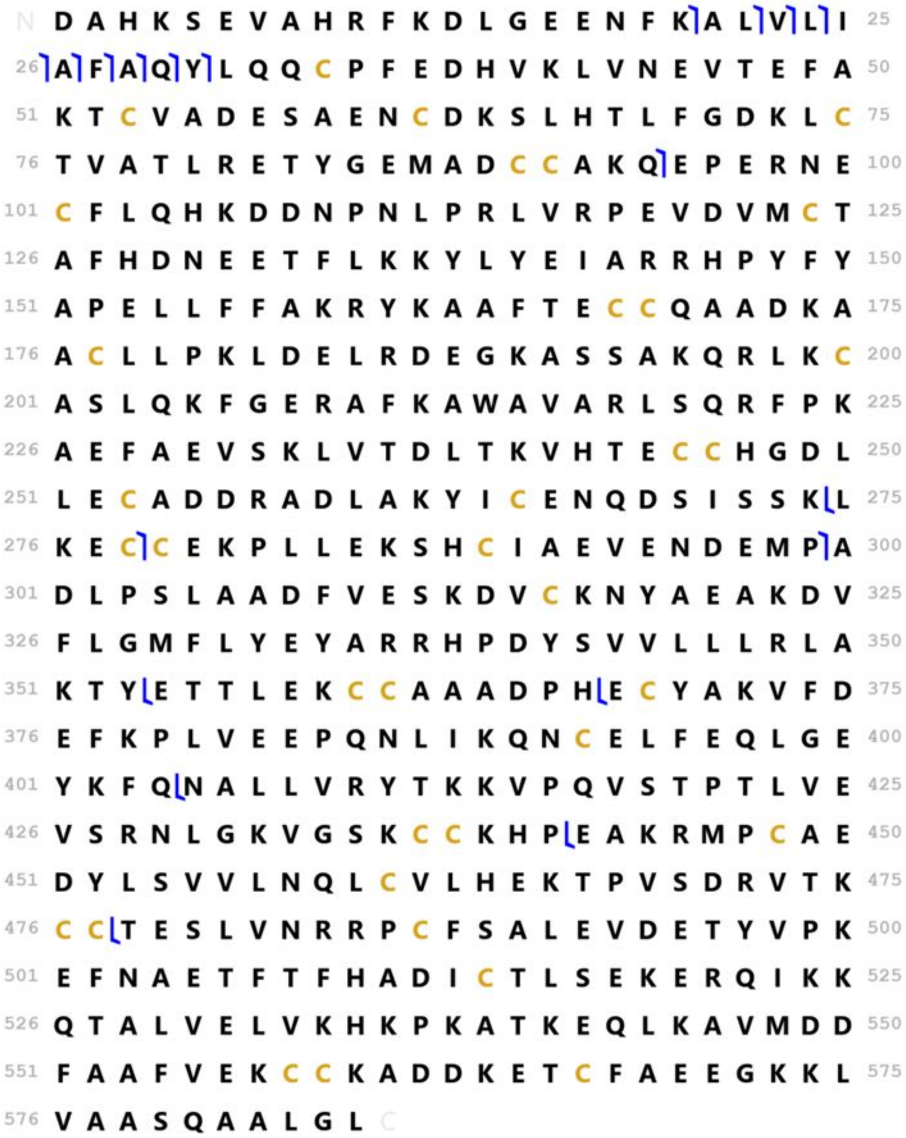

Figure S3. Sequence and fragmentation pattern of human serum albumin (HSA) from the CZE-MS/MS analysis ("low-high" mode) of the protein corona sample. The sequence of the mature form of HSA is shown without the signal peptide and the propeptide. No post-translational modifications (PTMs) and disulfide bonds were considered. The ProSight Lite software was used to match the experimental MS/MS data with the target protein sequence with a 50-ppm mass tolerance. The mass tolerance was determined based on the mass error of our instrument when the experiment was done.

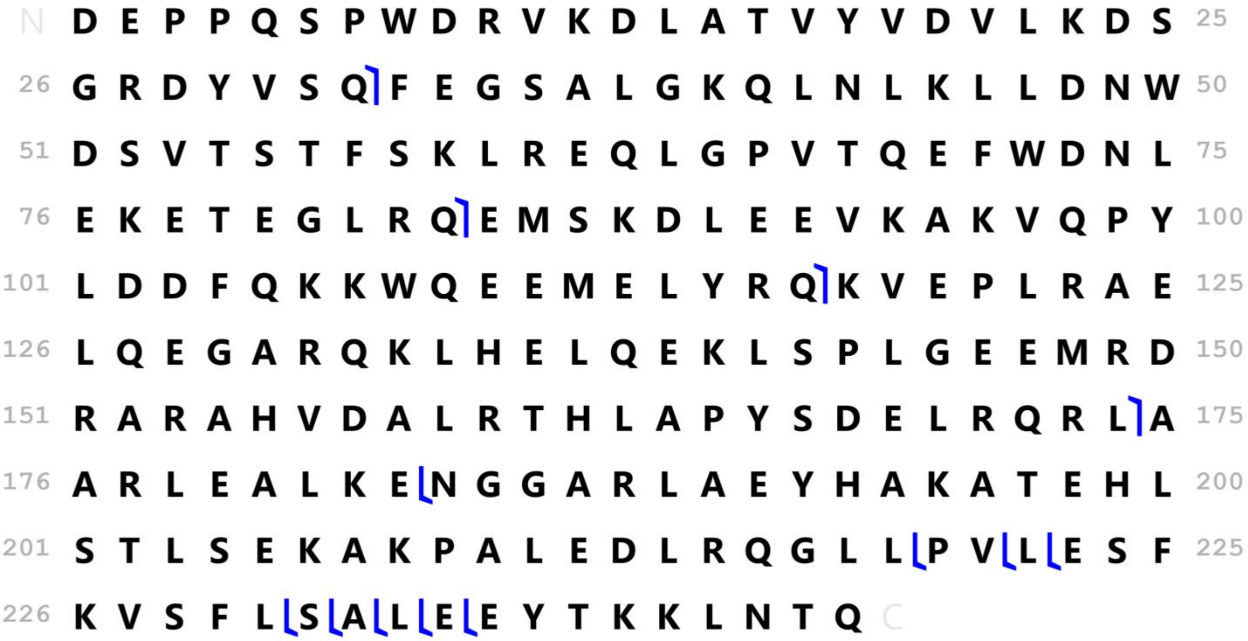

Figure S4. Sequence and fragmentation pattern of Apolipoprotein A-I (APOA1) from the CZE-MS/MS analysis (“low-high” mode) of the protein corona sample. The sequence of the mature form of APOA1 is shown without the signal peptide. No post-translational modifications (PTMs) were considered. The ProSight Lite software was used to match the experimental MS/MS data with the target protein sequence with a 50-ppm mass tolerance. The mass tolerance was determined based on the mass error of our instrument when the experiment was done.

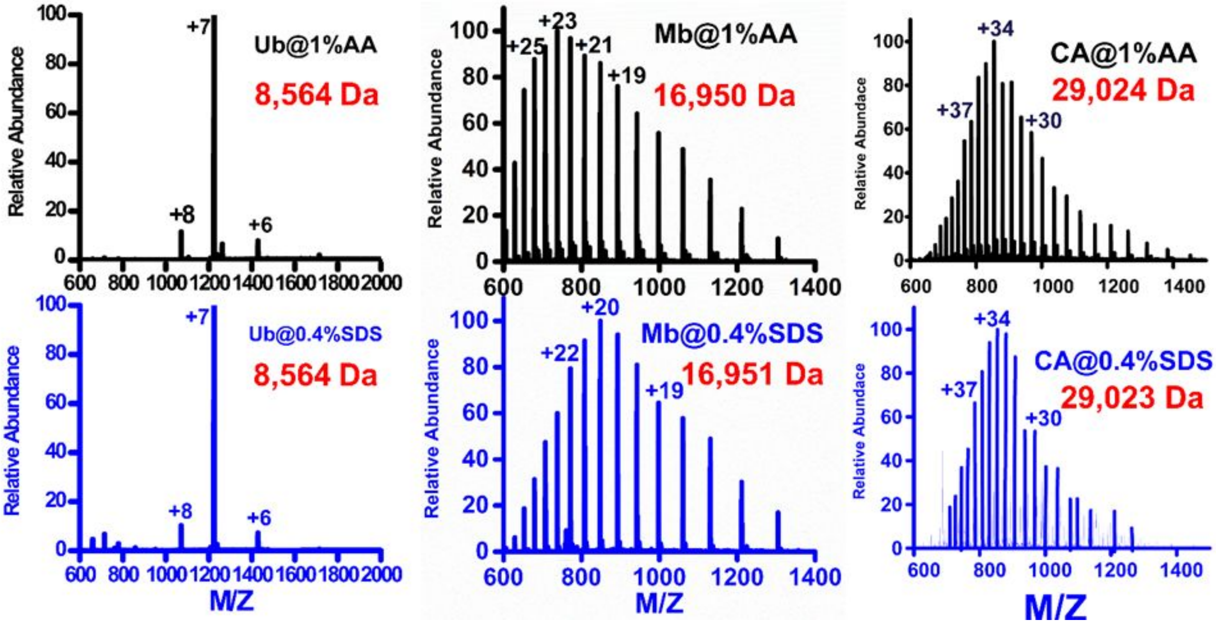

Figure S5. Mass spectra of a standard protein mixture in two different conditions. The first condition is that proteins are dissolved in 1% acidic acid (AA) for CZE-MS analysis (the black mass spectra, top). The second condition is that proteins are dissolved in 0.4% SDS, similar to the protein corona sample, followed by buffer exchange and CZE-MS (the blue mass spectra, bottom). Ub, Mb, and CA represent ubiquitin, myoglobin, and carbonic anhydrase, respectively. The data was acquired on an Agilent 6545XT Q-TOF mass spectrometer.

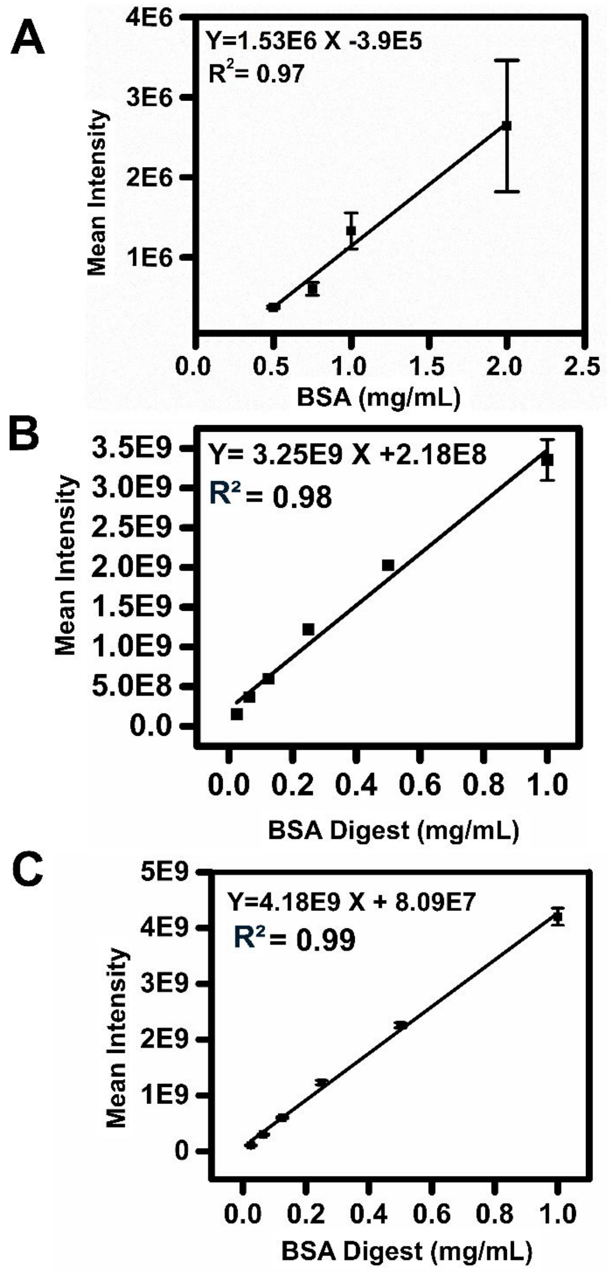

Figure S6. Calibration curve data of intact bovine serum albumin (BSA, 0.5-2 mg/mL, A) and two high-intensity peptides of BSA tryptic digests (0.025-1 mg/mL, B and C) analyzed by CZE-MS. Each sample was analyzed in triplicate. The mean of proteoform or peptide intensity from triplicate analyses was used as the y-axis. The error bars represent the standard deviations of proteoform or peptide intensity from triplicate runs.

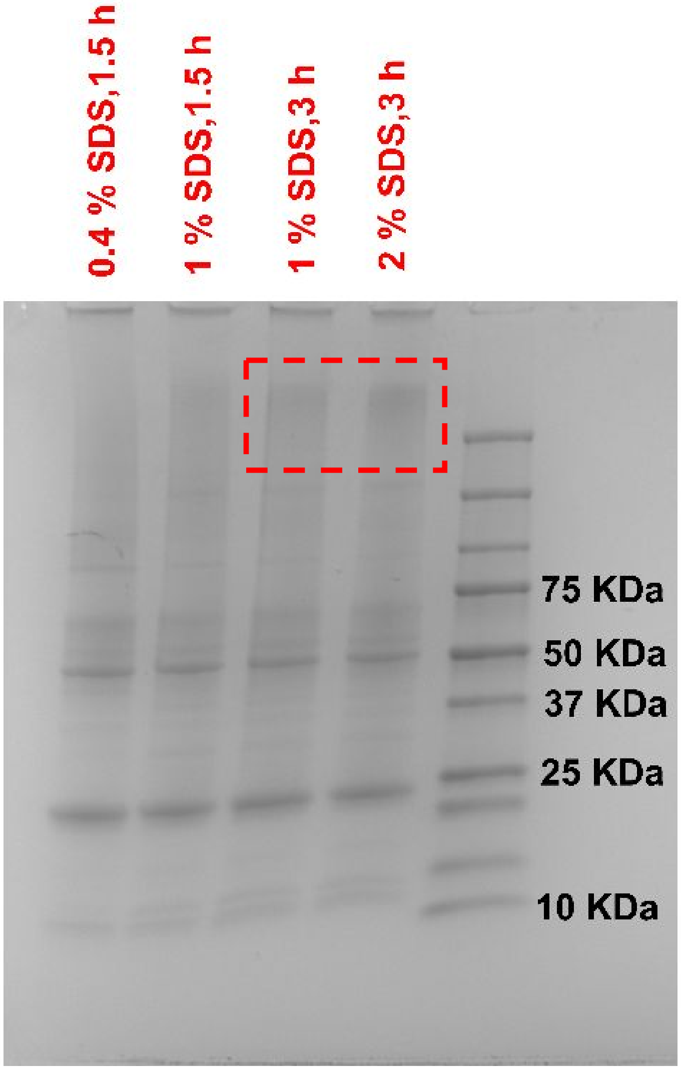

Figure S7. SDS-PAGE analysis of the eluted protein corona from PSNPs using different concentrations of SDS (0.4%, 1%, and 2%) and incubation time (1.5 h and 3 h).

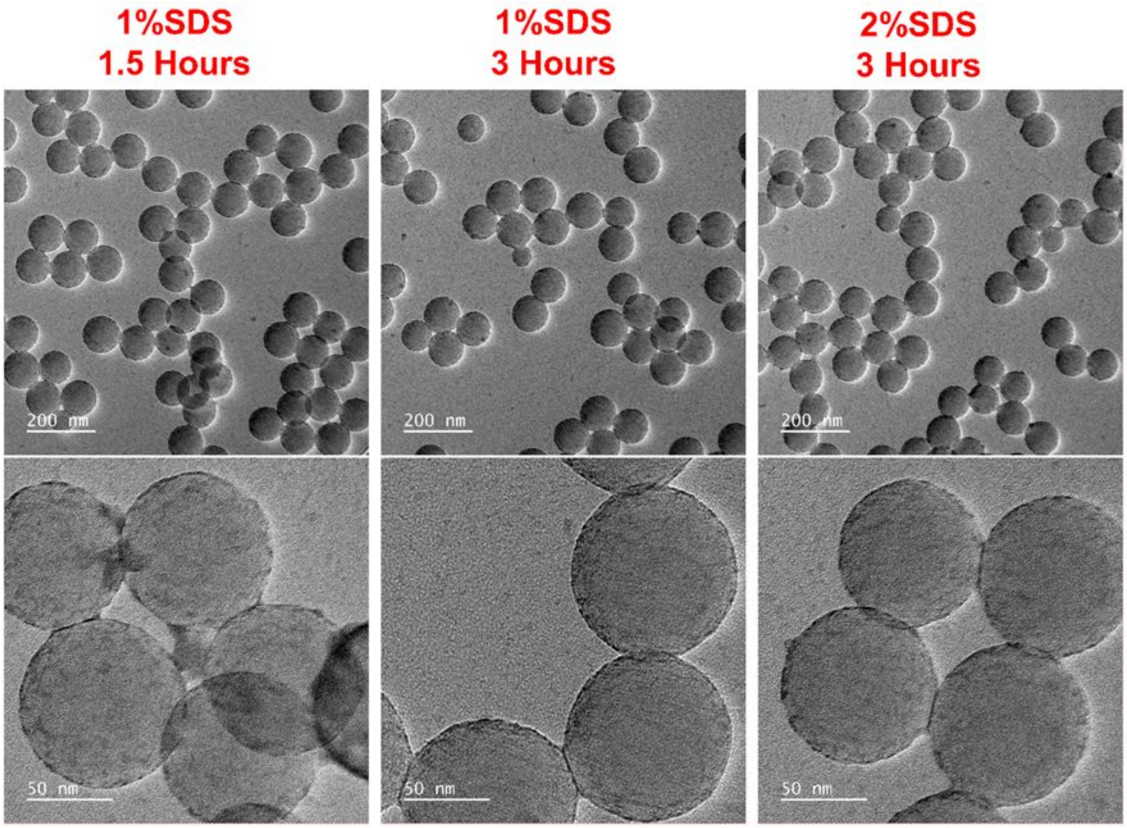

Figure S8. TEM images of protein corona-coated PSNPs after three different protein corona elution conditions, 1% SDS with 1.5 h incubation (left), 1% SDS with 3-h incubation (middle), and 2% SDS with 3-h incubation (right).
